# Home-based self-administered transcranial direct current stimulation for women affected by primary dysmenorrhoea in Northeastern Brazil: a protocol study

**DOI:** 10.1136/bmjopen-2025-100964

**Published:** 2025-07-13

**Authors:** Tatiana Camila de Lima Alves da Silva, Yvinna Tamiris Rodrigues, Edson Silva-Filho, Paloma Cristina Alves de Oliveira, Thiago Anderson Brito De Araújo, Ervinas Bernatavicius, Alexander Anthony Cook, Emilè Radytè, Rodrigo Pegado, Maria Thereza Micussi

**Affiliations:** 1 Graduate Program in Physical Therapy, Federal University of Rio Grande do Norte, Natal, Rio Grande do Norte, Brazil; 2 Graduate Program in Health Science, Federal University of Rio Grande do Norte, Natal, Rio Grande do Norte, Brazil; 3 Samphire Neuroscience Ltd, London, UK; 4 Graduate Program in Applied Sciences of Women's Health and Graduate Program in Physical Therapy, Universidade Federal do Rio Grande do Norte, Natal, Rio Grande do Norte, Brazil

**Keywords:** NEUROLOGY, Chronic Pain, Depression & mood disorders, Anxiety disorders, Quality of Life

## Abstract

**Abstract:**

**Background:**

The prevalence of women with primary dysmenorrhoea is high and negatively impacts physical and mental health. The intense cyclic episodes of pain generate central nervous system dysfunctional processing. In this sense, strategies focused on the central nervous system are important to re-establish normal functioning. Home-based self-administered transcranial direct current stimulation (tDCS) emerges as a strategy to modulate dysfunctional brain areas and improve the symptoms. This protocol aims to evaluate the effects of home-based self-administered tDCS for pain, premenstrual symptoms, physical performance, quality of life, electroencephalography and patient global impression in women affected by primary dysmenorrhoea.

**Methods and analysis:**

This is a single-centre, parallel, randomised, double-blinded clinical trial protocol. 40 women affected by primary dysmenorrhoea will be randomised into two groups (active-tDCS or sham-tDCS). Then, 20 consecutive sessions of home-based self-administered tDCS will be performed. The assessments will occur at five time points: baseline, after the 20th sessions, at the first, second and third cycles after tDCS interventions (follow-ups). Primary outcome will be pain according to visual analogue scale. Quality of life, premenstrual symptoms screening, depression, anxiety, physical performance, electroencephalography and participants’ satisfaction will be the secondary outcomes. A mixed analysis of variance will calculate the effect of stimulation.

**Ethics and dissemination:**

The study was approved by the ethics committee of the Federal University of Rio Grande do Norte (No. 6.037.756) and registered in the Brazilian Clinical Trials Registry (n° RBR-747k8vb). Participants may withdraw at any time without penalty. Free support will be available from the lead researcher if needed. All procedures will follow Good Clinical Practice and international ethical standards.

**Trail registration:**

https://ensaiosclinicos.gov.br/rg/RBR-747k8vb

**STRENGTHS AND LIMITATIONS OF THIS STUDY**

- Participants will independently operate the transcranial direct current stimulation (tDCS) device through a smartphone application.
- The intervention involves a long-term treatment protocol consisting of 20 consecutive tDCS sessions.
- Long-term outcomes will be assessed across five menstrual cycles.
- Findings may have limited generalisability beyond the studied population and regional context.

## Introduction

 Primary dysmenorrhoea is defined as painful menstrual cramps generated in the uterus,^1^ characterised by the absence of visible structural abnormality or any pelvic gynaecological disease.^2^ Despite primary dysmenorrhoea being one of the most common gynaecological conditions among women, especially adolescents, it is frequently undertreated.^1^ Affecting approximately 25% of adult women and up to 90% of adolescent women, primary dysmenorrhoea is associated with the prevalence of severe and disabling pain.^3^ The incapacitating symptoms reported by women range from 15% to 20%.^3^ Painful symptoms in the lower abdomen and lumbar region are often accompanied by nausea, vomiting, dizziness, headache, fatigue and diarrhoea.^3^ The pain onset usually exhibits a predictable temporal pattern, starting right before or at the beginning of menstruation, lasting approximately 8–72 hours. Moreover, the pain is more severe during the first or second day of menstruation.^3 4^ The presence of signs and symptoms impacts women’s schooling, professional and leisure activities and consequently their quality of life.^4^

Some evidence shows that women affected by primary dysmenorrhoea present systemic pain’s dysfunctional processing, due to intense and cyclic episodes of pain.^5 6^ Peripheral nociceptive signals generated by receptor organs during the menstrual period are amplified, causing an increase in neuronal excitability due to peripheral sensory pain stimuli.^5 6^ In fact, the presence of central alterations may be part of the primary dysmenorrhoea pathophysiology, which is now considered a central sensitivity syndrome, similar to other chronic conditions, such as fibromyalgia and tension headaches.^3 7^ While the increased sensitivity of pain receptors after trauma or infection usually returns to normal over time, in chronic pain, hypersensitivity is sustained and amplified by an extensive central neural network that includes the dorsal spinal horn, the limbic system and cortical structures.^8^ The limbic and sensory systems mediate the hypervigilance cycle for sensory stimuli from pelvic organs, leading to chronic pain.^8^

In this context, specific techniques that can modulate central nervous system activity may be useful for pelvic pain control, such as primary dysmenorrhoea.^3^ Women with primary dysmenorrhoea reveal significant alterations in the functional connectivity of the anterior cingulate cortex and disruptions in brain metabolism and pain modulatory systems.^3 5^ Additionally, brain regions closely linked to cognitive and emotional pain processing, such as the medial prefrontal cortex, posterior cingulate cortex and insula, exhibit anomalous functional and structural changes in otherwise healthy women experiencing primary dysmenorrhoea.^3^ It is feasible that women affected by primary dysmenorrhoea manifest altered cross-network connectivity and a disbalance between these systems.^3^

Transcranial direct current stimulation (tDCS) is a non-invasive neuromodulation technique that releases an electrical microcurrent applied through the scalp, inducing changes in cortical excitability, according to the stimulation parameters.^9 10^ tDCS is considered a neuromodulatory intervention that induces alterations in the excitability of the human cortex, promoting physiological effects that extend to physical and behavioural aspects.^11 12^ Home-based remote tDCS solutions were widespread and advocated during the social isolation imposed by the COVID-19 pandemic period, yet, it appears to be following a clinical trend.^13^ Requiring participants to attend research sites, hospital facilities or rehabilitation clinics for multiple treatment sessions is a logistically challenging process. For many patients, these factors may lead to increased expenses for transportation, travel time, food and missed work or school, ultimately reducing access to treatment.

Home-based self-administered tDCS is a safe and easy-to-use treatment strategy with low adverse effects that could be an easier strategy to adhere to and implement. Also, home-based self-administered tDCS might be another treatment option for the many women affected by primary dysmenorrhoea. To date, several remote supervision tDCS protocols have been performed, focusing on chronic pain, musculoskeletal and behavioural disorders.^14^,^16^

We hypothesise that tDCS targeting the dorsolateral prefrontal cortex (DLPFC) and primary motor cortex (M1) will enhance therapeutic outcomes.^17 18^ Targeting the left DLPFC and the M1 with tDCS may be effective for primary dysmenorrhoea due to their roles in pain modulation and affective processing.^17^,^19^ The left DLPFC is a key node in the cognitive-affective dimension of pain and is involved in top-down pain control, emotional regulation and the processing of pain-related distress, which are commonly altered in women with dysmenorrhoea.^17^ Meanwhile, M1 stimulation has been shown to activate descending inhibitory pain pathways and reduce the perception of pain by modulating thalamocortical circuits.^10^ Combined stimulation of these areas may enhance both affective and sensory pain control, providing a dual mechanism of action that is particularly relevant for the multifaceted nature of menstrual pain. These brain regions are key components of the pain neuromatrix and are frequently characterised by hyperactivation and dysregulation in individuals experiencing chronic pain.^20^ Previous studies reported beneficial effects of tDCS on anxiety, functional capacity and pain in women with primary dysmenorrhoea.^17^,^19^ The stimulation of the DLPFC and M1 constitutes an optimal neuromodulatory strategy for addressing the multidimensional symptomatology of primary dysmenorrhoea, including both nociceptive and affective components.^17^,^19^

Considering these assumptions, this study aims to evaluate the effects of 20 home-based self-administered tDCS sessions in women affected by primary dysmenorrhoea over pain, quality of life, premenstrual symptoms, depression, anxiety, physical performance, electroencephalography and participants’ satisfaction.

## Methods

### Study design

This is a protocol study of single-centre, double-blind, parallel, sham-controlled, randomised clinical trial. The study protocol adhered to the Guidelines for Standard Protocol Items: recommendations for Interventional Trials. The study starts in April 2024 and is expected to be completed in April 2026. The participants will be followed for 6 months.

### Recruitment and eligibility criteria

The recruitment will be voluntarily recruited through social media (Facebook and Instagram) and posters placed in different departments of the university. Prospective participants will receive detailed information regarding the study methodology, encompassing its objectives, allocation to either sham or active groups, assessment protocols and intervention timeline.

The study is currently in the recruitment phase, which began in April 2024, and is expected to continue through April 2026, following the established inclusion criteria: (1) diagnosis of primary dysmenorrhoea, defined according to the No. 345 Primary Dysmenorrhoea Consensus Guideline^1^; (2) aged 18–45 years; (3) regular menstrual cycle lasting 28 to 32 days; (4) report a pain score of ≥4 on the numeric rating scale (0–10); (5) not lactating; (6) no history of brain surgery, tumour or intracranial metal implantation; no presence of metal implants in the head (7); and (8) no history of chronic genitourinary infections, alcohol or drug abuse. Exclusion criteria include (1) history of dizziness or epileptic disease, (2) current pregnancy, (3) severe headache after more than two tDCS sessions and (4) severe dizziness or migraine following more than two tDCS sessions.

### Interventions

The entire treatment involves 20 consecutive sessions of home-based self-administered tDCS, one session per day. Initially, the participants will receive orientation about how to use the remote tDCS device. One physical therapist will instruct the participants about the tDCS protocol, download the application and demonstrate how to handle the device, including the position on the head, and operate it using the smartphone application. The Samphire App for IOS will be used. The training will also include the best practices for device preservation and conservation, such as leaving the tDCS in a safe place, and not over-exposing it to the sun or a heat source. Moreover, the researcher will inform the risks and guidelines related to potential expected adverse effects of use, which include redness at the site, itching where the electrodes are in contact with the skin, headaches after use, dizziness or nausea. The researcher will configure the smartphone application to be accessible in the participants’ native language (Brazilian Portuguese), with an option to switch to English. After training, participants will receive the device to use alone, according to instructions.

### tDCS

The Samphire Neuroscience tDCS will be used. The device has four rectangular sponges (5.0 cm × 2.0 cm and 2.5 mm thickness) that must be impregnated by the user with a saline (0.9% NaCl) solution before placing the tDCS on the head. The two anode electrodes will be placed in the region of the M1 and the DLPFC of the left cerebral hemisphere, at points C3 and FC5, respectively. The two cathode electrodes will be placed under the same regions of the right hemisphere, C4 and FC6, according to the international electroencephalography (EEG) 10/20 system. Each participant will self-administer it during 20 sessions for 20 minutes per session. In the active tDCS group, the current will ramp up from 0 to 2.0 mA over 30 s, and ramp down over 30 s, for a 20 min total session time. In the sham tDCS group, to ensure blindness, the current will ramp up to 2.0 mA over 30 s, then ramp down to 0.01 mA, over 30 s. This method produces the same mimic sensations, such as itching and tingling observed during active tDCS, enabling blindness.^21^ Both active and sham tDCS groups will use identical tDCS devices and use the same App. The session type will be determined by a previous randomised code input into the Samphire App. During tDCS use, participants will receive instruction to feel free and continue with their habitual routine.

### Outcomes

**Figure 1:**
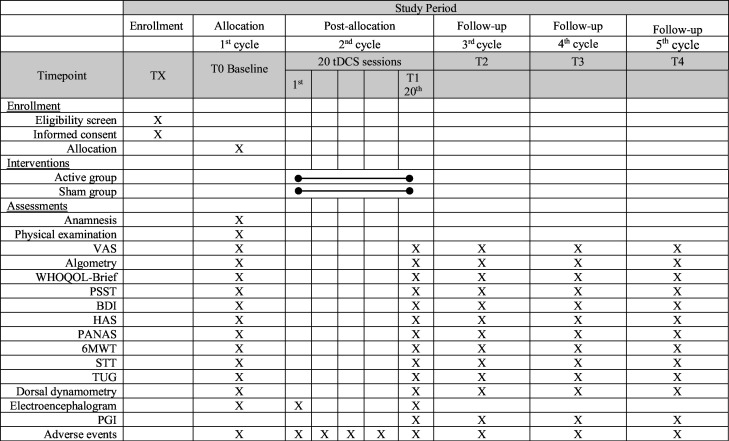
Schedule of enrolment, interventions and assessments. 6MWT, six-minute walk test; BDI, Beck Depression Inventory; HAS, Hamilton Anxiety Scale; PANAS, Positive and Negative Affect Schedule; PGI, Patient Global Intervention; PSST, Premenstrual Symptoms Screening Tool; STT, TUG, Timed Up and Go; VAS, visual analogue scale; WHOQOL, WHO Quality of Life.

The study will conduct evaluations at five time points, all scheduled to coincide with the onset of the first day of the menstrual cycle. The initial evaluation (baseline) will be conducted before any interventions occur, specifically on the first day of the participant’s first menstrual cycle. The second evaluation will occur after the completion of all 20 intervention sessions, specifically scheduled for the first day of the participant’s third menstrual cycle. Follow-up assessments will be conducted monthly, specifically on the first day of the participant’s third through fifth menstrual cycles. Figure 1 illustrates the evaluation process. The initial evaluation will include anamnesis, physical examination, pain assessment, and questionnaires.

The primary outcome is pain and will be assessed through the Visual Analogue Scale (VAS). The VAS is a unidimensional measure of pain intensity, consisting of a continuous scale composed of a horizontal line, 10 centimeters (100 mm) in length, anchored by two verbal descriptors, one for each extreme symptom.^22^ The scale is anchored by ‘no pain’ (score of 0) and ‘worst imaginable pain’ (score of 100 (100 mm scale)). Participants will self-complete the VAS, instructed to mark a line at the point representing the intensity of their pain during the day. The VAS serves as a direct pain index commonly used in clinical investigations of acute and chronic pain in patients, owing to its convenience and reliability.^22^

The secondary outcomes will include algometry, quality of life (WHO Quality of Life Questionnaire (WHOQOL-Bref)), premenstrual symptoms (Premenstrual Symptoms Screening Tool (PSST)), depression (Beck Depression Inventory (BDI)), anxiety (Hamilton Anxiety Scale (HAS)), affectivity (Positive and Negative Affect Scale (PANAS)), physical performance (6 min walk test, 30 s sit-to-stand test, Timed ‘Up and Go’ and dorsal dynamometry) and level of satisfaction with the intervention (Patient Global Intervention (PGI)).

A digital pressure algometer (MedDor, Brazil) will evaluate the pain sensitivity, observing the threshold and tolerance to pressure pain and the perception of pain intensity. The same trained examiner will position the pressure algometer perpendicular to the skin three centimetres below the umbilicus.^23^ Then, the examiner will place the 1 centimetre-diameter rubber tip over the examination area and gradually increase the pressure by approximately 1 kg/cm^2^/s to measure the pressure pain threshold and pressure pain tolerance.^23^ The examiner will describe the pain threshold when the patient first reports the beginning of pain perception through the phrase ‘start’. Additionally, for pain tolerance, the participant will be instructed to endure the maximum pressure applied by the algometer until reporting total inability to withstand such pressure, signalling with the phrase ‘stop’.^23^ Participants must repeat exactly these two sentences for complete standardisation of the exam. To measure the perception of pain intensity, the examiner will apply a painful mechanical stimulus for 5 s, considering the mid-point between the pressure pain tolerance and pressure pain threshold data, previously obtained from each participant.

The WHOQOL-brief will assess quality of life.^24^ This questionnaire presents 26 questions, with the initial two questions focusing on general quality of life. The remaining questions are divided into four domains: (1) physical domain includes facets, such as pain and discomfort, energy and fatigue, sleep and rest, mobility, activities of daily living, dependence on medication or treatments and ability to work; (2) psychological domain covers facets, such as positive feelings, thinking, learning, memory and concentration, self-esteem, body image and appearance, negative feelings, spirituality, personal beliefs and religion; (3) social relationships address facets, such as personal relationships, social support and sexual activity; and (4) environmental domain encompasses facets, such as physical safety and protection, home environment, financial resources, health and social care (availability and quality), opportunities to acquire new information and skills, participation and opportunity for recreation and leisure, physical environment and transportation. A higher score indicates a better quality of life.^24^ This questionnaire is a helpful tool to assess the impact of primary dysmenorrhoea on participants’ quality of life, social and personal functioning, known to affect overall well-being.^25^

PSS is a tool developed by Steiner *et al*,^26^ translated and validated to Portuguese by Câmara *et al.*^27^ This tool has 14 questions related to the presence and intensity of symptoms related to premenstrual syndrome. Also, the questionnaire presents five questions that investigate the interference of premenstrual syndrome in daily activities and individual relationships. All items include a Likert-type scale, ranging from 0 to 4, with 0 representing absence, 1 indicating mild, 2 denoting moderate and 4 indicating severe.^27^ A positive result for premenstrual syndrome requires the following options: the presence of at least five items from the first domain, classified from moderate to severe; at least one of the four main symptoms (anger/irritation, anxiety/tension, desire to cry/increased sensitivity to rejection, depressed mood/hopelessness) classified as moderate or severe; and at least one item from the second domain classified as moderate or severe. Participants who do not meet the above requirements are, according to the PSS tool, classified as having no premenstrual symptoms or having mild symptoms.^27^ This tool is important due to the high comorbidity of premenstrual symptoms in women affected by primary dysmenorrhoea.

BDI will assess depression levels. We will use a self-administered Portuguese-language questionnaire consisting of 21 items assessing symptoms and cognitive attitudes. For each item, the patient must choose one or more statements that best describe how they felt in the last week. The maximum score is 63 points, and higher scores indicate severe levels of depression. A score less than 10 points indicates no or minimal depression; from 10 to 18 mild to moderate depression; from 19 to 29 moderate to severe depression; and from 30 to 63 severe depression.^28^

HAS will evaluate anxiety. This was one of the first rating scales developed to measure the severity of anxiety symptoms and is still widely used today in clinical and research settings. The scale has 14 items, ranging from 0 to 4, with a total score of 56. The higher the score, the greater the degree of anxiety.^29^

The PNAS will assess affectivity. This questionnaire consists of a set of 20 words that describe different feelings and emotions felt by the patient during the last 60 weeks. This questionnaire has two dimensions, using 10 words to calculate positive affectivity and 10 for negative affectivity. Each word is scored from 1 (not at all) to 5 (extremely), and the total scores can vary from 10 to 50 for each dimension.^30^

The questionnaires WHOQOL-Bref, PSST, BDI, HAS, PANAS and PGI will be administered using versions that have been translated, culturally adapted and validated for use in Brazilian Portuguese.

Four tests will assess physical performance: the 6MWT, the 30 s sit-to-stand test, the TUG and dorsal dynamometry. The 6MWT is an evaluation of the maximum distance covered by the patient during 6 min. The 6MWT is widely used to assess submaximal functional capacity.^31^ During the test, there will be two standardised phrases to encourage the patient, in the third and fifth minutes, aiming at increasing their physical performance. The parameters measured before and after the test will be the respiratory rate, heart rate, haemoglobin oxygen saturation, Borg scale, presence of pain, and total distance covered.

The 30 s sit-to-stand test aims to measure the lower limbs’ strength. There will be a stopwatch and a chair with a height of 43 cm with a backrest. The patient will sit in the middle of the chair with feet flat on the floor and arms crossed over the chest. The score will correspond to the number of times the person performs the complete movement in 30 s.^32^

The TU test measures the lower limbs’ muscular strength and includes power, speed, agility and dynamic balance. It provides a comprehensive assessment of general muscle strength and physical function. We will instruct the subjects to stand up from the sitting position at the examiner’s signal, walk a distance of 3 m in a comfortable place, turn around, walk back to the chair and sit down again.^33^

Dynamometry will assess the isometric strength of the trunk extensor muscles. To perform the test, the participant will stand on the dynamometer platform with their knees fully extended but not hyperextended. Then, the trunk will flex forward, forming an angle of approximately 120° between the trunk and lower limb. The participants will position their heads in the midline with their gaze fixed straight ahead and their elbows extended.^34^ The dynamometer handle will be adjusted according to the size of the participant so that he/she can hold the support bar to maintain the position described above. The grab bar must be close to knee height. After that, participants will use their spine extensors to pull the handle towards their trunk as strongly as possible. The Crown 200 kg dynamometer will record the resultant force. There will be three attempts to generate the data. The highest value will be retained for analysis.^34^ At the end of the procedure, the pain perception of participants will be evaluated using the numeric rating scale, ranging from 0 to 10.

An EEG will assess cerebral effects and modification of electrical patterns. Brain electrical activity will be recorded using OpenViBE Acquisition Server (v3.2.0 - 64bit) and OpenViBE Design (v3.2.0 - 64bit) software, employing a 32-channel EEG system (engineering model actiCHamp, Brain vision), with a sampling rate of 10 kHz. Electrodes will be placed according to the international 10–20 system on an elastic cap (actiCAP DE-82211 model, EASYCAP GmbH), which will be comfortably adjusted on the participants’ heads. Each of the 32 electrodes will be positioned at specific locations corresponding to the following brain areas: FP1, FP2, AFp1, AFp2, AFZ, AF3, AF4, AF8, AF7 (GND), AFF1h, AFF2h, Fz, F1, F2, F3, F4, F5, F6, F7, F8, FC1, FC2, FC5, FC6, CZ, C3, C4, P3, P4, P7, P8, O1 and O2. To minimise impedance, a conductive paste (SuperVisc gel - 1000 g, EASYCAP GmbH) will hold the electrodes. Additionally, there will be a reference electrode at Fz. During recording, participants will remain seated and will be instructed to avoid any unnecessary movements. Recording will take place in a quiet, dimly lit room to minimise recording artefacts. A computer will save the data for offline analysis. To ensure data quality, the following measures will be adopted: (1) impedance will be monitored during electrode placement to ensure it remains below 5 kOhm; (2) before recording commences, the researcher will check electrical noises; and (3) during recording, the responsible researcher will continuously monitor signal quality and check for movement artefacts, eye blinking or muscle activity. EEG will be conducted at baseline (T0), after the first tDCS session (T1) and after 20 tDCS sessions.

The patient’s global impression will measure the participants’ satisfaction. It is a simple evaluation method composed of a seven-item scale, ranging from one (no changes) to 7 (much better). Improvement throughout the treatment involves the personal and psychometric aspects inherent to the pain treatment process. This scale identifies minor clinically important changes, which are sometimes not noticed in pain questionnaires or physical examinations.^35^

### Participant timeline

Figure 1 shows a general schedule, including enrolment, intervention and follow-ups. In the first month, the research proposal and randomisation will be explained, and the menstrual cycle will be monitored to identify the first day of the next menstrual cycle. In the second month, on the first day of the menstrual cycle, the participants will be assessed as baseline (**T0**) and will receive instructions and training about the use of home-based self-administered tDCS. In the third month, participants will start the tDCS sessions 20 days before the estimated date of the first day of the menstrual cycle. The assessment (**T1**) of all variables will be performed on the first day of the menstrual cycle. For three consecutive cycles after tDCS intervention, participants will be assessed as follow-up always on the first day of each menstrual cycle (**T2, T3 and T4**). A total of five assessments will be performed. It is important to note that there will be five evaluation time points, always on the first day of the menstrual cycle.

Before starting the trial, all researchers involved will be trained for evaluations and tDCS procedures. Five researchers will be involved in this clinical trial: one researcher for the evaluation procedures, two for the home-based self-administered tDCS instruction and training (instructor), one for the randomisation of participants and one for statistical analysis.

### Sample size

The sample size will follow previous research conducted by our group. The numerical rating scale for pain is the primary outcome of assessing women affected by chronic pelvic pain. The participants also attended five sessions of tDCS using a C3/Fp2 assembly.^19^ G*Power (V. 3.1.9.4, Düsseldorf, Germany) estimated the power and sample size requirements through the use of the F-test from a repeated measures analysis of variance, including the main effects of group and time and the two-way interaction between group and time. Based on an alpha of 0.05 and power of 0.80, two groups (active group and sham group), sphericity of 1 and effect size of 0.25, a total of 34 participants will be required, with 17 participants randomised to each group. With anticipated attrition, we will add six more participants, totalling 40 participants (active group, n=20; sham group, n=20).

### Randomisation, allocation concealment and blinding

A numerical sequence generated by appropriate software will assign each participant to the active tDCS group or the sham tDCS group, using a 1:1 allocation ratio. Each participant will be equally likely to belong to any group. An independent researcher not involved in the assessment or intervention will conduct all processes. Allocation concealment will be performed using opaque envelopes. Volunteers and researchers involved in assessments and interventions will maintain blinding to the participants’ allocation throughout the study.

Additionally, all procedures regarding the use of home-based self-administered tDCS will be performed for the sham group. First, the participants will position the device and turn it on. Then, they must follow the instructions and use the smartphone application. However, the tDCS will administer 2 mA of current with a ramp-up and ramp-down period of 30 s. Sham tDCS will involve administering active stimulation for a brief duration to replicate sensations (itching and tingling) observed during active tDCS.^21^

To initiate the tDCS protocol, participants will receive a code via email to access the smartphone application, allowing them to unlock 20 sessions of tDCS (one per day). The allocation of active or sham stimulation will be known only to a single researcher not involved in assessments or intervention.

### Statistical analysis

SPSS software version 21.0 (IBM Corp., Armonk, NY, USA) will analyse the data. We will describe the clinical sociodemographic characteristics using mean and SD for continuous variables and frequency and percentage for dichotomous variables. Independent t-test and X^2^ will analyse continuous and categoric sociodemographic data, respectively. Intention-to-treat analysis will include the data from participants who do not complete the entire treatment protocol. We will define the significance level in all statistical testing as a p value less than 0.05. After data collection, we will identify the best distribution to represent the data according to the residuals Q–Q plot and histogram. The generalised estimated equations or mixed model will analyse the data (primary and secondary outcomes at baseline, after the fifth session, after the last session and follow-ups). We will also evaluate if it is necessary to insert fixed or random factors into the constant based on A/C, B/C and Q/C indices, X^2^/DF and the intraclass correlation coefficient. The link function will estimate the results according to the distribution of the dependent variable. Time, group (active and sham groups) and the interaction between them will represent the independent factors. The mean, mean difference, SD, 95% CI and p value for baseline, after the last session and follow-ups will illustrate the results. Cohen’ d will represent the effect size.

### Harms

The researchers will carefully monitor adverse events in all steps of the study by asking patients, after each session of stimulation and during the follow-up periods, if they experienced any adverse events and the relationship of these events to treatment with tDCS. Any adverse event during the research period will be registered and considered for data analysis. The researchers will interrupt the sessions if any risk to the patients is suspected. To evaluate adverse effects, we will record any musculoskeletal symptoms, such as itching, tingling, burning sensation, headache, nausea and dizziness. Withdrawals will be excluded from the protocol and integrated as intention-to-treat analysis. Adherence to the number of missed sessions, number of dropouts and possible adverse events reported will be assessed.

### Ethics and dissemination

The Department of Physical Therapy of the Federal University of Rio Grande do Norte is the place where the researchers will collect the data. The study will follow the principles of the Declaration of Helsinki and Resolution 466/12 of the National Health Council. The researchers registered the protocol in the Brazilian Clinical Trials Registry (n° RBR-747k8vb) and obtained approval from the Federal University of Rio Grande do Norte Ethics Committee (n° 6.037.756).

Participants will retain the freedom to decline participation or withdraw their consent at any point during the study, without incurring any penalty or impact on their care. Should any issues arise during the trial, participants will have the right to receive free assistance from the designated responsible researcher. This study will not involve participants at risk of death or interventions that are harmful. Therefore, it will not be necessary to create a data safety monitoring board. All procedures will adhere strictly to international ethical and scientific standards, following the guidelines outlined in Good Clinical Practices for research involving human participants. The study findings will be communicated to participants through a public symposium and on social media.

The literature indicates that serious adverse events directly linked to tDCS are rare. All adverse events will be promptly reported to the principal investigator. Serious adverse events will be recorded and analysed by the study principal investigator and, if necessary, submitted for review by the Ethics Committee. No auditing procedures are planned; however, if required, any amendments to the research protocol will be submitted to the Ethics Committee for approval. On completion of the study, the datasets used will be made available to interested parties on reasonable request to the corresponding author.

### Patient and public involvement

All participants will be briefed on the trial’s objectives and procedures, emphasising voluntary participation. The researchers will inform the participants about the research procedures and will obtain a written Informed Consent Form signed by the participants before initiating the study. Following the study, all participants who received the sham tDCS stimulation will be invited to undergo the active tDCS. The principal investigator will store the data for 5 years in the Physiotherapy Department at the Federal University of Rio Grande do Norte. After this time, he will incinerate all the documents. This study will not share any personal information related to participation in the research during or after the study. The results of the study will be communicated to participants through a popular symposium.

## Discussion

This study protocol represents the pioneering use of a home-based self-administered tDCS approach for women suffering from primary dysmenorrhoea. The investigation will entail a comprehensive analysis of long-term treatment outcomes involving 20 consecutive sessions and a 3 month follow-up period. Primary dysmenorrhoea is recognised as a chronic pain condition that profoundly impacts women worldwide during their reproductive years.^5^ Moreover, individuals affected by primary dysmenorrhoea often present comorbid symptoms of depression, anxiety and affective disorders.^36^

During the COVID-19 pandemic, there has been a significant number of studies employing home-based self-administered tDCS or remote tDCS.^37^ This innovative approach is designed to enhance patient adherence, reduce costs and evaluate the effectiveness of remote neuromodulation treatments.^37^ Promising results have been demonstrated for remote tDCS treatment across different conditions. The introduction of new devices that show excellent usability, safety features, ease of use and interactive interfaces could greatly benefit a substantial number of patients, particularly those with significant impairments or facing transportation challenges.

The intermittent nature of pain experienced by women suffering from primary dysmenorrhoea, particularly exacerbated in the days leading up to and during menstruation, leads to brain reorganisation and central sensitisation.^5^ Neuroimaging studies have elucidated significant associations between pain and morphology as well as dynamic functional alterations in various brain regions implicated in the cognitive and affective regulation of pain.^5 6^ tDCS emerges as a neuromodulation technique aiming to improve pain in several chronic pain conditions. For tDCS, the most used target to modulate pain is the M1.^9^ Anodal stimulation on M1 increases the activity of this brain area and modulates the neural network by cortico-thalamic pathway.^38^

Other brain areas typically involved in the pain regulatory system also seem to be modulated, such as grey periaqueductal substance, cingulate cortex, indirectly the amygdala, primary and secondary S1.^38^ Prefrontal cortex is a second target usually studied for treating chronic pain.^38^ This is an executive area with a direct association with pain-related emotions, probably involved in the affective-emotional networks related to pain.^19 38^ Studies suggest that the DLPFC upregulates positive reactions to positive emotional stimuli and down-regulates negative reactions to emotional stimuli.^38^ This is important because women suffering from primary dysmenorrhoea show not only pain disturbance but also mood disorders including emotional distress, depression and anxiety.^1 7^ Physical symptoms are usually described and include low physical function or incapacitation before and during the menstrual period.^1 2^

This study protocol of home-based self-administered tDCS study aims to improve women’s health by assessing physical function, mood and electrophysiological aspects. The population proposal will involve only women affected by primary dysmenorrhoea. However, future studies could investigate other chronic pain conditions associated with women’s health that include interstitial cystitis, endometriosis and fibromyalgia.
