# Supplementary material for: Home-based self-administered transcranial direct current stimulation for women affected by primary dysmenorrhoea in Northeastern Brazil: a protocol study
Source: BMJ Open. 2025 Jul 13;15(7):e100964. doi: 10.1136/bmjopen-2025-100964 (PMC12258309; doi:10.1136/bmjopen-2025-100964)
Supplement: Supplementary file 1 [file bmjopen-2025-100964.reviewer_comments.pdf]

PEER REVIEW HISTORY

BMJ Open publishes all reviews undertaken for accepted manuscripts. Reviewers are asked to complete a checklist review form and are provided with free text boxes to elaborate on their assessment. These free text comments are reproduced below.

ARTICLE DETAILS

Title (Provisional)

Home-based self-administered transcranial direct current stimulation for women affected by primary dysmenorrhea in Northeastern Brazil: a protocol study

Authors

Silva, Tatiana Camila de Lima Alves da; Rodrigues, Yvina Tamiris; Silva-Filho, Edson; Alves de Oliveira, Paloma Cristina; Araújo, Thiago Anderson Brito De; Bernatavicius, Ervinas; Cook, Alexander Anthony; Radytè, Emilè; Pegado, Rodrigo; Albuquerque Barbosa, Maria Thereza

---

VERSION 1 - REVIEW

**Reviewer**	**1**
**Name**	**Inagawa, Takuma**
**Affiliation**	**National Center of Neurology and Psychiatry, Department of Psychiatry**
**Date**	**13-Mar-2025**
**COI**	**None**

Thank you for giving me the opportunity to review this manuscript.

This paper was a study protocol to assess the effects of tDCS over two sites on dysmenorrhea.

1) I think the rationale was not clear. Please describe why tDCS over the left DLPFC and M1 may be effective on dysmenorrhea. Were there any previous studies on the left DLPFC and M1dysfunction in patients with dysmenorrhea? Can tDCS improve those dysfunctions?

2) I think it is better to describe how to make sure that tDCS was truly placed over the specific areas. Were there any measures (teh MEG?) to assess this?

3) The authors described that the primary outcome was VAS to assess the intensity of the pain. On the other hand, there are so many previous studies on the effects of tDCS on pain. Please describe what the previous studies have been found, and what kind of tDCS protocol may be the best, and what is the novelty of this study to assess the effects of tDCS on pain.

I think it is better to revise the manuscript before publication.

**Reviewer**	**2**
**Name**	**Bayır, Buse Rahime Hasırcı**
**Affiliation**	**İstanbul Haydarpaşa Numune Eğitim ve Araştırma Hastanesi, neurology**
**Date**	**07-Apr-2025**
**COI**	**None**

A subject that many women have problems with, such as dysmenorrhea, has been addressed. The effectiveness of new methods such as tDCS in this area has been evaluated. It has been discussed in detail and has become an exciting article.

VERSION 1 - AUTHOR RESPONSE

Reviewer: 1

Dr. Takuma Inagawa, National Center of Neurology and Psychiatry
Comments to the Author: Thank you for giving me the opportunity to review this manuscript. This paper was a study protocol to assess the effects of tDCS over two sites on dysmenorrhea.

1) I think the rationale was not clear. Please describe why tDCS over the left DLPFC and M1 may be effective on dysmenorrhea. Were there any previous studies on the left DLPFC and M1dysfunction in patients with dysmenorrhea? Can tDCS improve those dysfunctions?

Response: Thank you for the suggestion. We have expanded the rationale in the revised manuscript (lines 99–120 and 139–144) to better explain the rationale for targeting the left DLPFC and M1 with tDCS in individuals with primary dysmenorrhea. Our research group has previously published three studies investigating this approach, all of which demonstrated positive outcomes using tDCS over the primary motor cortex and the dorsolateral prefrontal cortex in this population. These findings support the hypothesis that modulating these regions (known to be involved in pain perception, affective regulation, and descending pain inhibition) may help address the functional alterations observed in women with primary dysmenorrhea. References to these studies have been added to the manuscript.

2) I think it is better to describe how to make sure that tDCS was truly placed over the specific areas. Were there any measures (teh MEG?) to assess this?

Response: Thank you for the comment. The tDCS electrode placement in this study will follow the internationally recognized 10–20 EEG system, which has been widely validated and recommended as a reliable and accurate method for locating cortical targets in tDCS protocols. Several studies (e.g., 10.3791/2744, 10.1111/ner.12786) and expert guidelines (e.g., 10.1016/j.clinph.2017.06.001, 10.1016/j.brs.2020.02.011) support this approach as an effective alternative to more complex neuroimaging-guided techniques in standard research settings. The electrode positioning procedures are described in detail in the manuscript (lines 188–204). Given the study's pragmatic design and home-based application, the 10–20 system offers a balance between feasibility and precision for targeting the left DLPFC and M1. As the study

involves home-based tDCS application, it is not feasible to perform EEG or motor evoked potential (MEP) assessments during each stimulation session to confirm electrode positioning over the targeted areas. In general, participants will receive in-person training during the initial session to ensure proper device handling and correct electrode placement. Additionally, EEG recordings will be conducted before and after the full tDCS protocol; however, it will not be possible to perform EEG monitoring during each stimulation session due to clinical and logistical constraints. Magnetoencephalography (MEG) is not commonly used in tDCS studies because of its high cost, the need for specialized personnel, and limited practical applicability in this context. All of this information is described in the Methods section of the study.

3) The authors described that the primary outcome was VAS to assess the intensity of the pain. On the other hand, there are so many previous studies on the effects of tDCS on pain. Please describe what the previous studies have been found, and what kind of tDCS protocol may be the best, and what is the novelty of this study to assess the effects of tDCS on pain.

Response: Thank you for the comment. We have added relevant information to the manuscript (lines 133–146). To date, only two published studies have investigated the effects of tDCS specifically in women with primary dysmenorrhea, one targeting the primary motor cortex (M1) and the other targeting the dorsolateral prefrontal cortex (DLPFC). While tDCS has been studied in various chronic pain conditions, it is important to recognize that different types of chronic pain involve distinct mechanisms, such as nociplastic, neuropathic, or nociceptive pain. Therefore, evidence from one condition cannot be generalized across all chronic pain syndromes. To establish a reliable level of evidence and support clinical recommendations, more than two studies are needed, particularly for conducting systematic reviews and meta-analyses. Furthermore, the strength of recommendation and level of evidence for tDCS varies by condition—for example, tDCS has received Grade A evidence in favor of use for fibromyalgia, whereas it has received Grade A evidence against use for nonspecific low back pain. These discrepancies highlight the importance of evaluating the physiological and clinical effects of tDCS within each specific condition, rather than relying on generalized conclusions based on “chronic pain” as a whole. Our study contributes to addressing this gap by providing a condition-specific, rigorously designed protocol focused on primary dysmenorrhea. As noted, primary dysmenorrhea is considered a neglected condition, and further studies exploring the effects of tDCS and other neuromodulation techniques are necessary to strengthen the level of evidence and improve treatment recommendations for this population.

Reviewer: 2

Dr. Buse Rahime Hasırcı Bayır, İstanbul Haydarpaşa Numune Eğitim ve Araştırma Hastanesi
Comments to the Author: A subject that many women have problems with, such as dysmenorrhea, has been addressed. The effectiveness of new methods such as tDCS in this area has been evaluated. It has been discussed in detail and has become an exciting article.

Response: We sincerely thank Dr. Buse Rahime Hasırcı Bayır for her positive and encouraging comments. We are pleased that the relevance of primary dysmenorrhea and the potential role of innovative approaches such as tDCS were recognized. We appreciate your kind words regarding the level of detail and the overall contribution of the manuscript, and we hope that this study will help advance research and clinical interest in this important, yet often under-addressed, condition.

VERSION 2 - REVIEW

Reviewer 1

Name Inagawa, Takuma

Affiliation National Center of Neurology and Psychiatry, Department of Psychiatry

Date 13-Apr-2025

COI

Thank you for giving me the opportunity to review this manuscript.

I think this manuscript would be suitable for publication.
